# Postoperative infections after oesophageal resections: the role of blood transfusions

**DOI:** 10.1186/1477-7819-4-80

**Published:** 2006-11-21

**Authors:** Francesca Rovera, Gianlorenzo Dionigi, Luigi Boni, Andrea Imperatori, Alessandra Tabacchi, Giulio Carcano, Mario Diurni, Renzo Dionigi

**Affiliations:** 1 Department of Surgical Sciences, University of Insubria, Varese, Italy

## Abstract

**Background:**

Perioperative blood transfusion carries numerous potential risks concerning the transmission of infective diseases and immunodepression that can facilitate the occurrence of postoperative infectious complications. Explanation of connections between perioperative blood transfusion and postoperative septic complication worldwide is not well documented. Many studies have described a correlation between perioperative blood transfusions and postoperative infections. On the contrary, other studies indicate that factors influencing the need for blood transfusions during surgery have a greater bearing than blood transfusion per se on the occurrence of postoperative complications.

**Patients and methods:**

A prospective study was conducted in our Department on 110 consecutive patients undergoing oesophageal resection for primary cancer, in order to evaluate the incidence of postoperative infections related to perioperative allogenic blood transfusions. For each patient we preoperatively recorded in a computerized data-base several known risk-factors for postoperative infections; in detail we registered the administration of allogenic perioperative blood transfusions (period of administration, number of packages administered).

**Results:**

Among the enrolled 110 patients, 53 (48%) received perioperative blood transfusions: in this group postoperative infections (overall infective complications) occurred in 27 patients. After a multivariate analysis we observed that perioperative blood transfusions significantly affected as an independent variable the development of wound infections (p = 0.02).

**Conclusion:**

Blood transfusions independently affected the incidence of wound infections in patients who underwent oesophageal resection for primary cancer.

## Background

Nosocomial infections are the most frequent complications observed in surgical oncological patients; despite considerable progress in the areas of prevention, diagnosis and therapy, postoperative infections continue to be associated with significant morbidity, sometimes with mortality and with extra expense to cover cost of antibiotics, blood derivatives, nursing, additional surgical procedures and prolonged hospitalization [1-3].

Most surgical infections are acquired intraoperatively and are endogenous, arising from the flora of the patient's skin, gastrointestinal tract or mucous membranes. Exogenous infections are less common and are probably acquired from the skin or nasal flora of the operating team, or more rarely from contaminated material or instruments in the operating theatre.

The risk of postoperative surgical infections is mainly related to the magnitude of surgical field contamination. The opening of the lumen of the organ containing bacteria always involves a relevant risk for postoperative wound infections. In 1964, Altemeier and the National Research Council [4] proposed a classification of surgical procedures related to the degree of bacterial contamination. This wound classification scheme has served as the basis for recommending antibiotic use, for preoperative bowel preparation, for directing wound management and for focusing wound surveillance.

Surveillance studies using classification of surgical procedures related to operative field contamination showed large variations in postoperative infection rates among different Centres, for the same type of procedures. These discrepancies led to the identification of other risk factors that might significantly influence the occurrence of infectious complications. It is through the control of such factors that better clinical results may be achieved.

These other factors affecting the incidence of postoperative infections have been identified through clinical and experimental studies carried out during the last decades; these may be divided into two groups: treatment-related and patient-related. The first group includes quality of surgical care, type and duration of surgery, emergency procedure, immunosuppressive therapy, blood transfusions. The second group comprises advanced age, co-morbidity, pre-existing infections, malnutrition and host defence deficiency.

Perioperative blood transfusion carries numerous potential risks concerning the transmission of infective diseases and immunodepression that can facilitate the occurrence of postoperative infectious complications. Explanation of relation between perioperative blood transfusion and postoperative septic complication is not well documented.

Many studies have described a correlation between perioperative blood transfusions and postoperative infections [5-10], suggesting that blood transfusion interferes with the immune system of the recipient; thus transfusion-related immunomodulation may have an impact on host defence and on the clinical course of patients who received blood components. Firstly, the immunosuppressive effects of allogeneic blood were noticed in 1973, when it was shown that renal transplant survival was improved in a group who received blood transfusions. Since then, several clinical studies, comparing groups of patients which needed perioperative blood transfusions with non-transfused patients, were carried out to evaluate the real immunosuppressive mechanism and its relationship with postoperative infectious complications. Moreover, studies evaluating the correlation between the amount of transfused blood units with infective complications demonstrated dose-related effect [11,12].

On the other hand, other studies indicate that factors influencing the need for blood transfusions during surgery have a greater bearing than blood transfusion *per se *on the occurrence of postoperative complications [13,14]. In fact, those clinical studies underlying the relationship between blood transfusion and postoperative infective complications have difficulties in being adjusted for the effects of many confounding variables related to the severity of illness, the various risk-factors for infections at specific sites and the surgical procedure.

Nevertheless, a meta-analysis recently published and some larger observational studies, in which Authors tried to make adjustments for many potentially confounding variables, came to the conclusion that this correlation exists [15-17].

Prospective studies investigating the association between blood transfusions and postoperative infections are needed, to justify more accurately the indications for blood transfusions.

### Aims of the study

A prospective study was conducted in our Department of Surgical Sciences of University of Insubria in Varese, between August 1987 and January 2005, on 110 consecutive patients undergoing oesophageal resection for primary cancer. The purposes of this prospective study were to set up a data bank on surgical infections observed, in order to systematically register such infections complications; to observe the incidence of postoperative infections related to perioperative blood transfusions.

## Patients and methods

We prospectively studied 110 consecutive patients admitted to our Institute and undergoing resection for oesophageal cancer performed by the same surgical team. The average age was 62 ± 9 years (range 38–80); 92 were male and 18 female.

For each patient, we preoperatively recorded in a computerized data-base the following risk-factors: diabetes, steroid therapy, obesity, chronic renal failure, cirrhosis, pre-existing chronic pulmonary disease, neoadjuvant radio-chemotherapy, nutritional and immunological parameters such as serum albumin and lymphocyte count.

Postoperatively, we prospectively monitored patients daily recording the development of infective complications such as pneumonia, wound infection, urinary tract infection, as well as septic complications and the presence of abdominal abscess and anastomotic leakage.

The type of operation, the duration of surgery, the preoperative hospitalization, the need of antibiotic therapy, the postoperative hospital stay were also registered. A careful monitoring and registration of perioperative blood transfusions (number of units and period of administration) was done.

According to the classification proposed by Altemeier and the National Research Council, in the absence of pre-existing thoracic or abdominal infection and if the surgical procedure is carried out with skilled technique, the oesophageal resection is considered a clean-contaminated procedure, because there is a surgical opening of the oesophagus and subsequent anastomosis, with controlled contamination of the surgical field.

### Analysis of results and statistical methods

The significance of any difference in the characteristics of the oesophageal cancer patients was evaluated. Where differences were found Cox's proportional hazard regression (univariate and multivariate analysis) was used to identify independent predictors of postoperative infections.

Data were expressed as mean with standard deviation or median and range according to the data distribution. Student's t test was used for analysis of continuous data and the chi-square test was used to compare differences between proportions. Mann-Whitney U Test was used to compare median values. P-values < 0.05 were considered significant. Statistical package for social sciences (SPSS version 11.0, MapInfo Corporation, Troy, NY, USA) was used for data analysis.

## Results

After resection of the oesophageal cancer, surgical reconstruction was obtained by oesphago-gastric stapled anastomosis in 107 patients (97%; 20 cervical and 87 thoracic), oesophago-digiunal anastomosis in 2 patients and an anastomosis with pharynx in 1 patient.

Out of 110 patients, 12 (11%) had wound infections, 19 (17%) pulmonary infections; 10 (9%) urinary tract infections; 15 (14%) intra abdominal abscess and anastomotic leakage. The perioperative mortality was 2.7% (3 patients). Table 1 shows biochemical data related to the development of surgical infections.

**Table 1:** Association between continuous variables and postoperative infections

	Patients who developed infections (N = 47) (Mean ± SD)	Patients who did not develop infections (N = 63) (Mean ± SD)	p-value
Haemoglobin (g/dl)	13.5 ± 2	13.5 ± 2	NS
Haematocrit (%)	39.8 ± 4.8	40.6 ± 5.6	NS
Serum albumin (g/dl)	3.9 ± 0.4	3.9 ± 0.7	NS
Blood lymphocytes level/mm^3^	1766 ± 764	1869 ± 790	NS
Preoperative hospital stay (days)	15.8 ± 12.6	12 ± 7	0.057
Postoperative hospital stay (days)	15.8 ± 12.6	17.9 ± 12.7	NS
Length of operation (min)	300 ± 87	276 ± 99	NS

Among the enrolled 110 patients, 53 (48%) received perioperative blood transfusions, with an average number of blood units transfused per patient of 1.5 ± 2 (range 1–12). In all these patients blood transfusions were administered perioperatively (intraoperatively or in the first 48 hours from surgical procedure). In the transfused group postoperative infections (overall infective complications) occurred in 27 (51%) compared to 20 (35%) of infections registered in 57 non-transfused patients. In a univariate analysis this difference did not reach a statistical significance: p = 0.093 (Table 2).

**Table 2:** Incidence of postoperative infections in transfused and non transfused patients (univariate analysis)

	Patients who received transfusions (n = 53)	Patients who did not receive transfusions (n = 57)	p-value
# patients with a postoperative infection	27	20	0.093
Wound infections	8	4	0.175
Respiratory infections	10	9	0.670
Urinary tract infections	9	1	0.005
Abscess/anastomotic leakage	9	6	0.324

After a multivariate analysis we observed that perioperative blood transfusions was an independent variable for the development of wound infections (p = 0.02), while the development of postoperative infection, pulmonary infections, urinary tract infections and anastomotic dehiscence with abscess were not significantly influenced by blood transfusions as independent factor (p-value 0.11; 0.93; 0.13 and 0.82 respectively). The microorganisms frequently isolated from different cultural samples are detailed in tables 3, 4 and 5.

**Table 3:** More frequent microorganisms isolated from infected wound

Type of microorganism	# patients
*Escherichia coli*	8
*Staphylococcus aureus*	6
*Pseudomonas aeruginosa*	3
*Enterococcus faecium*	2
*Staphylococcus epidermidis*	2
*Enterococcus faecalis*	1

**Table 4:** More frequent microorganisms isolated from sputum in patients with respiratory infections

Type of microorganism	# patients
*Pseudomonas aeruginosa*	12
*Staphylococcus aureus*	9
*Candida species*	5
*Escherichia coli*	3
*Enterobacter cloacae*	2
*Stenotrophomonas maltophilia*	2
*Staphylococcus epidermidis*	1
*Haemophilus influenzae*	1
*Enterococcus faecalis*	1

**Table 5:** More frequent microorganisms isolated from urine in patients with urinary tract infections

Type of microorganism	# patients
*Enterococcus faecalis*	2
*Enterococcus faecium*	2
*Escherichia coli*	2
*Staphylococcus aureus*	1
*Enterobacter cloacae*	1
*Staphylococcus epidermidis*	1
*Candida species*	1

## Discussion

In the last decade the immunosuppressive effect of blood transfusion has been studied in detail.

During the early nineties a large number of patients who underwent major surgical procedures received blood transfusions, several studies were carried out to investigate whether homologous blood transfusions significantly affect postoperative septic morbidity and mortality [18]. Clinical effects of transfusion-induced immunosuppression in surgical patients have been largely debated in the literature with contradictory results.

The immunosuppressive effect is supported when considering the lower degree of allogeneic transplant rejection and a higher frequency of cancer recurrence in patients who underwent blood transfusions [19].

Historically, Opelz and Terasaki demonstrated that blood transfusions before surgery of kidney transplant may prolong graft survival. Likewise, it was noticed that blood transfusions seemed to prevent recurrent abortions and led to lower recurrence rates in Crohn's disease and, nowadays, several studies support the hypothesis that allogeneic blood transfusion is an independent risk-factor for the development of postoperative bacterial infection [20,21]. Transfusion-induced immunosuppression is thought to mediate this effect [22,23].

Three mechanisms are mainly involved in blood transfusions associated with infections: 1) direct transmission of the infection from donor to recipient through blood micro-organisms; 2) activation of pre-existant or latent recipient infection; and 3) activation of recipient immunity defence.

Immunological alterations following blood transfusion can produce a clinical effect.

Blood transfusion may interfere with the immunological response interacting with macrophages and with lymphocytes. These cells play a primary role in wound and anastomotic healing in the postoperative period, and raise the incidence of peri-anastomotic abscess and generalized peritonitis [24,25].

In fact it is documented that blood transfusions may delay wound and anastomotic healing by interfering with the specific actions of these cells. Moreover, blood transfusions in patients who underwent nephrodyalisis blocked the lymphocytic response toward different mitogenic and antigenic molecules; suppressor lymphocytes were enhanced and an inverted ratio between T-helper and T-suppressor lymphocytes was observed [26].

Prolonged T- lymphocytes suppression after blood transfusion in patients with chronic inflammatory bowel disease was reported [27]. Lymphocytic response to phitohaemo-agglutinin, concavalin A and such antigen was seen for a period of 4 weeks following blood transfusion in abdominal and thoracic surgery.

Blood transfusion has also been identified as a high risk-factor of complications in emergency surgery for bowel perforation related to abdominal blunt trauma [5]. The direct relationship between the number of blood units transfused and better survival was also documented [28-30].

Also the timing of administration seems to be relevant. Several Authors documented that the immunosuppressive effect of blood transfusion is greater when it is given intraoperatively or in the immediate postoperative period (within the first 48–72 hours from surgical procedure). Bellantone *et al*., reported a likely association between postoperative infections and postoperative transfusion [31].

Despite the above mentioned clinical studies seem to correlate blood transfusion as an independent risk-factor to postoperative complications, a causal relationship between blood transfusion and an increase in the risk of postoperative infection is hard to prove in the clinical setting, firstly because obviously these are not randomized studies and secondly for the doubtful role of too many confounding variables. It is based on these considerations that some authors proposed that the detection of a relationship between postoperative infections and perioperative blood transfusions may depend on many patient and surgical variables that may complicate interpretation of the results; indeed, it remains to be elucidated whether blood transfusion is an independent variable causing surgical infections, or it is a variable dependant on other more important risk-factors (i.e. anemia) [13].

After a careful multivariate analysis we observed a higher number of postoperative wound infections in patients that received perioperative blood transfusions compared with patients that did not (p = 0.02). Eight out of twelve patents (67%) who developed this kind of postoperative complication received blood transfusions. Blood transfusions independently affected the incidence of wound infections in patients who underwent oesophageal resection for primary cancer.

## Conclusion

The results of our study are similar to those obtained by other studies that came to the same conclusions we support a cautious interpretation of the effects of perioperative blood transfusions on postoperative infective complications. The blood transfusion may induce immunomodulation and consequently immunodepression so whenever possible perioperative blood transfusion has to be limited.

## Competing interests

The author(s) declare that they have no competing interests.

## Authors' contributions

**FR **and **AT**: acquisition of data, **GD, MD **and **AI**: study conception and design, **GC **and **LB**: analysis and interpretation of data, **FR**: drafting of manuscript, **RD**: critical revision and supervision
